# Novel Metabolic Abnormalities in the Tricarboxylic Acid Cycle in Peripheral Cells From Huntington’s Disease Patients

**DOI:** 10.1371/journal.pone.0160384

**Published:** 2016-09-09

**Authors:** Nima N. Naseri, Joseph Bonica, Hui Xu, Larry C. Park, Jamshid Arjomand, Zhengming Chen, Gary E. Gibson

**Affiliations:** 1 Weill Cornell Medical College, Brain and Mind Research Institute, Burke Medical Research Institute, 785 Mamaroneck Avenue, White Plains, NY 10605, United States of America; 2 Weill Cornell Medical College, Division of Biostatistics and Epidemiology, Department of Healthcare Policy and Research, 425 East 61st Street, New York, NY 10065, United States of America; 3 CHDI Management/CHDI Foundation, Inc., 6080 Center Drive. Suite 100, Los Angeles, CA 90045, United States of America; University of Texas Health Science Center at San Antonio, UNITED STATES

## Abstract

Metabolic dysfunction is well-documented in Huntington’s disease (HD). However, the link between the mutant huntingtin (mHTT) gene and the pathology is unknown. The tricarboxylic acid (TCA) cycle is the main metabolic pathway for the production of NADH for conversion to ATP via the electron transport chain (ETC). The objective of this study was to test for differences in enzyme activities, mRNAs and protein levels related to the TCA cycle between lymphoblasts from healthy subjects and from patients with HD. The experiments utilize the advantages of lymphoblasts to reveal new insights about HD. The large quantity of homogeneous cell populations permits multiple dynamic measures to be made on exactly comparable tissues. The activities of nine enzymes related to the TCA cycle and the expression of twenty-nine mRNAs encoding for these enzymes and enzyme complexes were measured. Cells were studied under baseline conditions and during metabolic stress. The results support our recent findings that the activities of the pyruvate dehydrogenase complex (PDHC) and succinate dehydrogenase (SDH) are elevated in HD. The data also show a large unexpected depression in MDH activities. Furthermore, message levels for isocitrate dehydrogenase 1 (IDH1) were markedly increased in in HD lymphoblasts and were responsive to treatments. The use of lymphoblasts allowed us to clarify that the reported decrease in aconitase activity in HD autopsy brains is likely due to secondary hypoxic effects. These results demonstrate the mRNA and enzymes of the TCA cycle are critical therapeutic targets that have been understudied in HD.

## Introduction

Mitochondrial dysfunction and oxidative stress are associated with Huntington’s disease (HD), a late-onset, neurodegenerative disease that causes severe motor dysfunction and death of select neurons in the brain. HD is an autosomal dominant inherited disease that is caused by an excessive number of polyglutamine (CAG) repeats in the huntingtin gene (HTT) [1].

Approximately 5.70 per 100,000 people are affected by this fatal disease [2]. The link between the genetic mutation and the neurological sequale is unknown. Mutant Htt (mHtt) forms aggregates in the nucleus of affected neurons, but the exact role and nature of these protein aggregates is still poorly understood [3].

Considerable evidence suggests that mitochondrial dysfunction links the genetic mutation to the neurological sequelae. Multiple FDG-PET studies reveal that glucose metabolism is decreased in the caudate nuclei and putamen of brains from HD patients [4–6]. Brain lactate is increased in HD, suggesting decreased metabolism through the tricarboxylic acid (TCA) cycle [7]. In addition, increased production of reactive oxygen species (ROS)[8], altered mitochondrial fission [9–11] and fusion [10,11], and changes in mitochondrial trafficking [12] accompany HD. Co-immunoprecipitation analysis using cortical protein lysates from HD patients reveals that mHtt interacts with the mitochondrial protein Dynamin-related protein 1, an important regulator of mitochondrial repair and biogenesis [13].

The main pathway for converting glucose to reducing equivalents (NADH) for production of ATP is the TCA cycle. Glucose is converted to pyruvate, and the oxidative decarboxylation of pyruvate to acetyl CoA by the pyruvate dehydrogenase complex (PDHC) is the entry step into the TCA cycle. The remainder of the cycle consists of the following enzymes in order: citrate synthase (CS), aconitase, isocitrate dehydrogenase (ICDH), α-ketoglutarate dehydrogenase complex (KGDHC), succinyl thiokinase (STH), succinate dehydrogenase (SDH), fumarate hydratase (FH) and malate dehydrogenase (MDH). The TCA cycle is highly integrated so that just measuring one enzyme does not give the full impact of the disease on the TCA cycle or the impact of the change on the disease process [14]. Some enzymes of the cycle have been measured in HD in separate studies, including PDHC [15], KGDHC [16], SDH [17], aconitase [18], and CS [18]. We recently measured the entire cycle along with PDHC [19] in the same set of HD autopsy brain samples and in the Q175 knock-in HD mouse model [20], and the current studies are designed to complement those results. Molecular analysis of autopsy tissue is compromised by many factors including autolysis, post-mortem interval, effects of drugs that the patient may have been taking at the time of death and severe degeneration and atrophy of tissues. The validity of animal models also presents serious compromise. The goal of this study was to determine if the TCA cycle is altered in HD at the mRNA, protein or specific activity levels in peripheral cells from HD patients that are not subject to these compromises. Observations in peripheral cells can serve as a window into CNS pathology, and possibly serve as a biomarker for future clinical trials. In addition, mRNA for transketolase, the rate-limiting enzyme of the pentose shunt, was determined as a preliminary consideration for evaluating the generation of NADPH.

Experiments were performed with cultured human lymphoblasts from healthy subjects and HD patients. The selection of lymphoblasts was justified for several reasons. First, huntingtin protein is found in peripheral cells [21]. Measuring the variables of interest in this study in peripheral tissue has many advantages. Lymphoblasts provide a readily available tissue source from HD patients and controls that contain the patients’ genetic material. Any possible effects of drugs or therapies are minimized by multiple passages in culture. Existing repositories of HD lymphoblasts allow for the selection of homogenous cell populations in which many critical variables can be controlled including age, sex and clinically relevant CAG repeat lengths. Furthermore, lymphoblasts can be rapidly expanded in culture to make many measurements under exactly the same conditions. They can be rapidly frozen to assure mRNAs are not degraded. The main compromises are that they are transformed, and they are not neurons. Thus, results with lymphoblasts provide a valuable complement to our studies of autopsied human brains and mouse brains.

Stress is often required to reveal compromised metabolism in cells or in the brain. For example, brains of mice deficient in KGDHC show minimal phenotypic response. However, their responses to 3-nitroproprionic acid or malonate (two chemical models of HD) are greatly exaggerated [22,23]. In this study, cells were stressed in two ways. First, cells were deprived of serum. Removing serum results in fewer trophic factors, plus the added advantage of better controlled conditions because components of FBS are poorly defined and may vary from one batch to another. The second stressor was the addition of cyanide, an irreversible inhibitor of complex IV of the oxidative phosphorylation chain [24], to cells in serum-free media. The variables of interest were determined under normal basal conditions, following serum deprivation and cyanide treatment following serum-deprivation.

## Methods

### Selection of lymphoblasts

All procedures received IRB approval by the Burke Rehabilitation Hospital IRB; BRC 293. Lymphoblasts were obtained from EHDN, Universitätsklinik Ulm, Abt. Neurologie Oberer Eselsberg, 89081 Ulm, Germany. Eight lines from healthy controls and eight lines from HD patients were selected based on sex, age, functional scores [20], and CAG repeat lengths (Table 1). Participants gave informed written consent according to the International Conference on Harmonisation-Good Clinical Practice (ICH-GCP) guidelines, and the study was conducted in accordance with the Declaration of Helsinki. For participants who lacked capacity to consent, study sites adhered to country-specific guidelines for obtaining consent. For data protection and confidentiality, all participants were assigned a unique pseudonym that does not contain any identifying information. Ethical approval was obtained from the local ethics committee for each study site contributing to REGISTRY [25]. The EHDN Executive Committee approved the study.

**Table 1:** Patient criteria for selecting lymphoblast cultures.

Group	Sex (M:F)	Age (yr)	CAG repeat	Functional Score
Control	4:4	44.3 ± 3.0	17.5 ± 1.4	
HD	5:3	43.8 ± 1.3	44 ± 0	12.8 ± 0.5

Healthy (control) and HD cell lines were selected based on sex, age and length of CAG repeats. Functional scores, determined by the Unified Huntington’s Disease Rating Scale (UHDRS), provide a standardized clinical assessment of HD that incorporates motor assessment, cognitive assessment, behavioral assessment, independence scale, functional assessment, and total functional capacity [20]. Values are means ± SEM (n = 8).

### Tissue culture maintenance

Frozen ampoules from EHDN were stored at -80°C. The cells were thawed in a 37°C water bath and grown in culture using Advanced RPMI 1640 + 2 mM L-GlutaMAX + 25 mM HEPES (Gibco, Grand Island, NY) + 15% FBS (qualified, US Origin) from Life Technologies (Carlsbad, CA) at 37°C (5% CO_2_). Multiple ampoules of 5 million cells each in 80% Advanced RPMI 1640, 15% FBS and 5% DMSO (Sigma, St. Louis, MO) were cryopreserved at passages 2–8 at -80°C.

The lymphoblasts were initially seeded at 8x10^5^ cells/ml in horizontally-laid T25 flasks and grown to no more than 1.5x10^6^ cells/ml. At this point, cells were expanded at 2x10^5^ cells/ml in horizontally-laid T75 flasks (CytoOne, USA Scientific, Ocala, FL) and grown for 3.5 days before passaging. These growth conditions were meticulously optimized based on Coriell Cell Repositories’ (Camden, NJ) recommendations. Cell lines were discontinued before 20 passages.

An arbitrarily chosen single healthy control line was paired with an arbitrarily chosen single HD line. Triplicate flasks were cultured for each treatment. These two lines were never compared to each other, but always as a part of the whole group.

### Treatment paradigms for lymphoblast cultures

Cells were grown to approximately 10^6^ cells/ml. For each cell line, three flasks were maintained in serum-containing media and six were maintained in serum-free media. The serum was removed by washing the cells once with Advanced RPMI 1640 + 2 mM GlutaMAX without serum and cells were subsequently resuspended in the same serum-free media. Cells were allowed to grow and equilibrate for another 48 hours. Sodium cyanide (1 mM final) was added to serum-free flasks and incubated for 6 hours. Thus, the three groups consisted of serum containing control flasks (54 hours), serum-free flasks (54 hours), and serum-free flasks (48 hours) plus the addition of NaCN (1 mM final) for six hours.

### Live/dead analysis

Aliquots for cell counts were taken at the 54 hour time point (described above). In the initial experiments, aliquots of the cells were stained with trypan blue, which only stains dead cells. Live and dead cells were then counted manually under a standard light microscope. Most experiments were done by staining aliquots of the cells with calcein AM (2 μM) and ethidium homodimer-1 (4 μM) from Life Technologies for 30 minutes at 37°C. Photographs were taken with a Nikon 80i Fluorescent Microscope (Tokyo, Japan). Calcein AM stains live cells green under fluorescent lighting. Ethidium homodimer-1 is impermeable to live cells and fluorescently stains dead cells red. The ITCN function in Image J from NIH was used to automatically count large numbers (1000^+^) of live and dead cells. The results were nearly identical using either method to count cells (data not shown).

### Flash-freezing cells for analysis

The cells were flash-frozen at the final time point of 54 hours and stored at -80°C for later molecular analysis. Cells were pelleted, washed once with D-PBS and then re-pelleted before flash-freezing them in liquid nitrogen. This flash-freeze method did not alter enzyme activities compared to unfrozen cells for any of the nine major enzymes of interest for this study (data not shown).

### Measurements of mRNA levels

Flash-frozen aliquots of lymphoblast cells were stored long-term (1–6 months) at -80°C. RNA was extracted using the RNeasy Mini Plus Kit from Qiagen (Venlo, Limburg). RNA concentrations and detection of potential protein impurities were measured using a NanoDrop 1000 spectrophotometer (Thermo Scientific, Waltham, MA). The High Capacity Reverse Transcription Kit with RNase Inhibitor (Life Technologies) was used to synthesize cDNA.

Human primers were obtained from Life Technologies. Real-Time PCR was run using TaqMan Fast Universal PCR Master Mix, No AmpErase UNG from Life Technologies using a 7500 Fast RT-PCR machine from Applied Biosystems (Carlsbad, CA): 20 seconds at 95°C; 40 cycles for 3 seconds at 95°C and 30 seconds at 60°C, total volume equals 20 μl. Thirty different endogenous controls were screened and carefully tested. The three (HPRT1, GAPDH, and CDKN1A) with the least amount of variation between treated and untreated samples were compared before selecting HPRT1 as the final internal control. Each target gene (Table 2) was measured in triplicate on each of the three flasks per treatment group.

**Table 2:** Genes associated with the enzymes of the TCA cycle, PDHC and transketolase.

Enzyme	Gene	Description/function	Control Gene Expression Relative to HPRT1
Transketolase	TKT	Thiamine-dependent enzyme which may play an important role in the pentose-phosphate pathway.	1.73 ± 0.27
Pyruvate Dehydrogenase Complex	DLD	Converts dihydrolipoamide to lipoamide	1.88 ± 0.06
	DLAT	Transfers an acetyl group to CoA	0.866 ± 0.077
	PDHX	Non-catalytic subunit	0.771 ± 0.051
	PDHB	Two alpha and two beta subunits for E1 heterotrimer	0.896 ± 0.061
	PDHA2	Two alpha and two beta subunits for E1 heterotrimer Found primarily in spermatogenic cells	0.001 ± 0.0003
	PDHA1	Two alpha and two beta subunits for E1 heterotrimer	1.58 ± 0.15
	PDK2	Phosphorylates E1 alpha subunit. Highest expression is in heart/skeletal muscle. Intermediate expression in brain.	0.034 ± 0.008
	PDK4	Located in matrix. Phosphorylates E1 alpha subunit. Ubiquitous. Highest expression in heart and muscle.	0.0005 ± 0.0002
	PDP1	Dephosphorylation of E1. Predominantly exists in skeletal muscle.	0.006 ± 0.0009
Citrate Synthase	CS	Catalyzes synthesis of citrate from oxaloacetate and acetyl CoA.	1.96 ± 0.13
Aconitase	ACO2	Catalyzes interconversion of citrate to isocitrate via cis-aconitase.	0.443 ± 0.022
Isocitrate Dehydrogenase	IDH1	Found in the cytoplasm and peroxisomes.	0.123 ± 0.020
	IDH2	Found in mitochondria.	1.75 ± 0.19
	IDH3G	Gamma unit of heterotrimer (2 alpha, 1 beta, 1 gamma subunit).	1.55 ± 0.14
	IDH3B	Beta unit of heterotrimer (2 alpha, 1 beta, 1 gamma subunit).	0.354 ± 0.040
	IDH3A	Alpha unit of heterotrimer (2 alpha, 1 beta, 1 gamma subunit).	2.22 ± 0.01
α-Ketoglutarate Dehydrogenase Complex	DLD	Converts dihydrolipoamide—> lipoamide.	1.88 ± 0.06
	DLST	Catalyzes succinyl-CoA to CoA.	0.747 ± 0.108
	OGDH	Decarboxylates alpha-KGA to form succinyl CoA.	1.17 ± 0.12
Succinyl Thiokinase	SUCLG2	Catalyzes the GTP dependent ligation of succinate and CoA to form succinyl-CoA.	0.474 ± 0.034
	SUCLG1	Catalyzes conversion of succinyl CoA and GDP to succinate and GTP.	0.853 ± 0.048
	SUCLA2	Hydrolyzes ATP to convert succinate to succinyl-CoA.	0.300 ± 0.043
Succinate Dehydrogenase	SDHD	Oxidizes succinate by carrying electrons from FADH to CoQ.	0.790 ± 0.129
	SDHC	Anchors other subunits of the complex to the inner membrane.	0.165 ± 0.015
	SDHB	Oxidizes succinate by carrying e- from FADH to CoQ.	1.37 ± 0.17
	SDHA	Accepts and transfers electrons from succinate to SDHB.	1.71 ± 0.27
Fumarase	FH	Hydration of fumarate to malate.	2.51 ± 0.19
Malate Dehydrogenase	MDH1	Localized to cytosol. Assists movement of malate through mitochondrial membrane to be transformed into oxaloacetate.	1.88 ± 0.03
	MDH2	Protein localized to mitochondria.	2.80 ± 0.30

The 29 genes listed were selected based on their roles in the 9 enzymes that were examined and the role of transketolase in the pentose shunt. Most of these genes either code for subunits of the enzymes or for proteins that play integral roles in their functions (e.g. the kinases for PDHC). Mean gene expressions relative to HPRT1 ± SEM are listed for healthy subjects under basal serum conditions (n = 7).

### Protein measurements

Protein levels were measured using a Bradford Coomassie brilliant blue dye-binding procedure (Bio-Rad 500–006 kit; Bio-Rad Laboratories, Hercules, CA) [26]. Bovine Serum Albumin (BSA) from Sigma-Aldrich was used as the reference standard.

### Enzyme activity measurements

Enzyme activity measurements were made in lysates from frozen lymphoblast samples. Each assay was standardized to a commercial enzyme from Sigma Aldrich to minimize betweenrun variance. Enzyme activities were measured and calculated as previously described [19].

### Western blot analysis

The proteins were loaded onto Novex Tris-Glycine 4–20% gels (Life Technologies EC6025) and then transferred overnight onto nitrocellulose paper (Bio-Rad Laboratories, cat. # 262–0115). Membranes were blocked with Li-COR Odyssey Blocking Buffer (Li-COR Biosciences, Lincoln, NE, cat. # 927–4000) diluted in Tris Buffered Saline (TBS). Antibodies from Abcam (Cambridge, UK) were anti-SDHB (SDH) [cat. # ab14714], mitoProfile pyruvate dehydrogenase WB Antibody Cocktail (PDHC), anti-pyruvate dehydrogenase (PDH) cocktail (cat. # ab110416) for the following proteins: 69 kDa E2, 54 kDa E3, 43.3 kDa E1[alpha], 39.4 kDa E1[beta], anti-aconitase 2 (cat. # ab71440), anti-MDH2 (MDH, cat. # ab96193) and antiICDH (cat. # ab113232). STK19 primary antibody (STH, cat. # Sc-28027) was purchased from Santa Cruz Biotechnology (Dallas, TX). Primary β-actin rabbit monoclonal antibody from Cell Signaling Technology (Beverly, MA) or mouse β-actin primary polyclonal antibody (Cell Signaling, cat. # 4970S) were added to detect β-actin. Secondary goat anti-rabbit IgG (Li-COR, cat. # 926–68071) and secondary goat anti-mouse antibodies (Li-COR, cat. # 926–32210), were added to detect actin and the protein of interest. The membranes were scanned using the LiCOR Odyssey instrument.

### Statistical analysis

#### qPCR calculations

Each treatment was done in triplicate. Each gene in each flask was measured in triplicate (essentially yielding triplicates of triplicates). The differences of C_t_ values between the target gene and the reference gene (ΔC_t_), HPRT1, were used for statistical analysis. To account for the correlation among the triplicates within each sample, GEE method assuming exchangeable correlation structure was used to find the statistically significant differences between groups (ΔΔC_t_). The fold change in between groups was then calculated using the group difference (2^ΔΔCt^) [27]. The correction for multiple testing was not carried out due to the exploratory nature of this study. All tests are two-sided with p ≤ 0.05 to declare statistical significance. All analyses were performed with the use of statistical software SAS9.3 (SAS Institute, Cary, NC).

#### Enzyme activity calculations

Enzyme standards from Sigma were used to establish a linear range of activity tracings before every assay. Each sample was measured in triplicate and then averaged. Outliers were eliminated using a q test at 80–85% confidence [28]. These values were then averaged with the other samples of the same group. The response to serum-deprivation and to serum-deprivation plus cyanide for each cell line was assessed for significance at p≤0.05 using a paired, two-sided Student’s t-test. Furthermore, differences between the healthy and HD lines under basal serum, serum-free, and serum-free plus cyanide conditions were determined using ANOVA at p≤0.05.

#### Calculations for Western blots

Membrane scans were evaluated and quantified using Odyssey 3.0 software (Li-Cor Biosciences). Band intensity values were measured for the enzymes of interest and the reference standard, β-actin. β-actin intensity values were normalized to the highest β-actin band signal. The ratio of enzyme band intensity to normalized β-actin band intensity was averaged for each subject group. Changes after treatment and differences between healthy and HD lines were compared under serum, serum-free, and serum-free plus cyanide conditions. Significance was quantified at p≤0.05 using a two-sided Student’s t-test.

## Results

### Sodium cyanide exacerbated cell death in healthy controls but not in HD cells after initial serum deprivation

**Fig 1:**
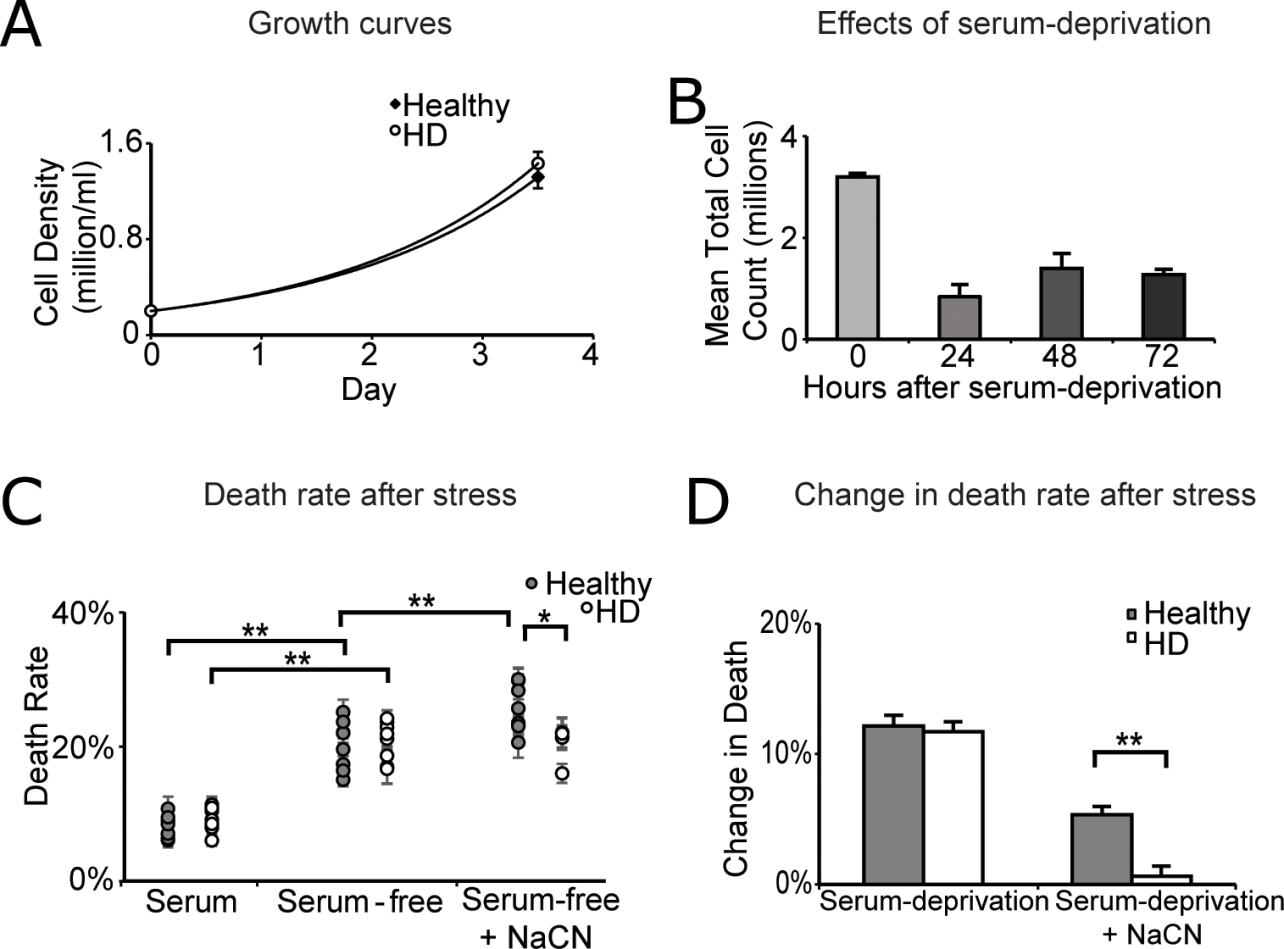
HD cells respond uniquely to metabolic stress. (A) Growth rates were established and compared during the initial stages of the study. Cells were always seeded at 2x10^5^ cells/ml and left to grow for 3.5 days before passaging. Two healthy and two HD cell lines were counted 14 times each (n = 28) using a hemocytometer. Live cell counts were approximately equal between healthy (1.32x10^6^) and HD (1.43x10^6^) lines after 3.5 days of growth. (B) Lymphoblasts were seeded as described in the ‘Tissue culture maintenance’ section in serum-free media. The total number of live cells was monitored every 24 hours for 72 hours (n = 3). The error bars represent standard errors of the mean. (C) Death of lymphoblasts under serum, serum-free, and serum-free plus cyanide conditions. Cell death was assessed as described in the ‘Live/dead analysis’ section. Cell death was measured in triplicate per flask and then averaged. Each experiment consisted of three independent flasks. The average cell death of the three flasks together was determined to be the percent death for that specific cell line for that particular experiment (n = 1). The treatment was repeated 3–4 more times per cell line. Thus, the cell death for each cell line for each treatment was determined to be the average of 3–4 experiments (essentially triplicate or quadruplicate experiments of identical triplicate flasks, each of which was measured in triplicate). This process was repeated for all 16 cell lines (n = 8 healthy, n = 8 HD). Values are the means ± SEM. The average death rates for each individual cell line is plotted to show consistency of trends. (D) The average change in cell death induced by removal of serum for 54 hours and additional death induced by addition of 1 mM NaCN for 6 hours after 48 hours of serum-deprivation. The average cell death for each cell line was determined as described in Fig 2A. The difference in cell death between serum and serum-free conditions was calculated based on these averages for each cell line. The deltas in death for all 8 cell lines were then averaged to determine the final average change in cell death induced by removing serum from the media for each subject group (healthy and HD). The same process was repeated for the cyanide treatment. *p≤0.05 determined by Student t-test. **p≤0.01 determined by Student t-test.

The growth rates of the healthy and HD cell lines under standard tissue culture conditions in the presence of serum were comparable (Fig 1A). Thus, any differences cannot be attributed to abnormalities in growth. Treatment conditions were established to stress the lymphoblasts. Serum-deprivation was used as the first of two stressors. As expected, a large number of cells died after being deprived of serum (Fig 1B). By 24 hours, the number of living cells declined by about 70%. In spite of the continued high cell death (data not shown), the cell number did not decline further up to 72 hours after serum-deprivation, indicating that a stable state had been established. Sodium cyanide was added at a final concentration of 1 mM at 48 hours, during this stable state, for six additional hours. Doses of cyanide between 0.05–4 mM were tested from 1–30 hours (data not shown). Doses higher than 1 mM induced too much death to be useful for molecular analysis, whereas doses lower than 1 mM did not reproducibly elicit differences in cell death between the healthy and HD lines.

The responses to both serum-deprivation and sodium cyanide were remarkably consistent across cell lines and within sample groups (healthy versus HD) [Fig 1C]. As expected, the death was low in serum-containing media (8–9%). Serum-deprivation increased death in both the HD and healthy groups similarly (20–21% mean cell death). However, the response of the healthy and HD lines to cyanide differed: mean cell death increased from 20% to 25% in the lines from healthy subjects whereas it did not change in the HD lines (Fig 1D).

### Select mRNA levels consistently differed between healthy and HD lines with or without serum and cyanide

This study focused on 29 genes that are integral to the PDHC, the eight major enzymes of the TCA cycle and transketolase (TKT). Expression levels for these mRNAs were measured relative to HPRT1 (Table 2). Measuring each sample three times independently, each time in triplicate, allowed for accurate determination of small changes in gene expression with high confidence. Although many statistically significant changes with treatment and between HD and healthy subjects were identified, only the changes that were greater than 20% on average in either direction are discussed. Confidence intervals at 95% are listed (Table 3). All differences in Table 3 are statistically significant except panel C column 3.

**Table 3:** Comparison of mRNA in healthy and HD lymphoblasts and their responses to serum deprivation and cyanide treatment (95% confidence interval). All changes are significant (p≤0.05) except for in the third column of panel C.

**A**			
Gene	HD serum /Healthy serum		
PDHA1	+5% to +38.3%		
IDH1	+48.6% to +118.9%		
DLST	-37% to -1.9%		
SDHB			
SDHC	+8.4% to +45.1%		
**B**	**Serum-deprivation Response**		
Gene	Healthy serum-deprivation /Healthy serum	HD serum-deprivation / HD serum	HD serum-deprivation /Healthy serum-deprivation
TKT		+17% to +63.2%	
PDP1	+14.5% to +67.7%	+3.3% to +50.5%	
PDK4	+53.7% to +606.2%	+60.2% to +300%	
PDK2	+46.5% to +82.4%	+53.6% to +123.5%	
PDHA1			+14.6% to +41.4%
PDHA2	+14.9% to +151.4%		
IDH3A	-40.8% to -2.5%		
IDH3G		+14.3% to +45.4%	
IDH2	-41.3% to -8.6%		
IDH1	+69.2% to +165.7%	+25.1% to +117.3%	+6.5% to +85.3%
DLST			
SDHB			+4.4% to +64.9%
SDHC	+23% to +73.3%	+22.8% to +64.5%	+6.3% to +38.8%
			
**C**	**Cyanide Response**		
Gene	Healthy cyanide / Healthy serum-deprivation	HD cyanide / HD serum-deprivation	HD serum-deprivation + NaCN /Healthy serum-deprivation + NaCN
PDK4	-59.8% to -32%	-43.2% to -19.6%	
PDK2	-35.6% to -20.5%	-34.3% to -20.5%	
PDHA1			-26.4% to +10.5%
PDHA2	+3.3% to +72.4%		
IDH1		-64.5% to -33.5%	-4.3% to +13.4%
DLST			-3.2% to +20.8%
SDHB			-25% to +29.8%
SDHC			-3.7% to +3.1%

The statistical analysis for the qPCR is described in the ‘qPCR calculations’ section. Only changes that were greater than 20% on average in either direction are presented. The listed ranges are 95% confidence intervals significant at p≤0.05 or greater. All of the values that are shown are significant except for the third column of panel C. n = 7 cell lines (one pair of lines was not measured). (A) The fold difference in HD patients compared to healthy subjects under basal growth conditions. (B) The fold change response of healthy and HD cells to serum deprivation, and fold differences between HD and healthy cells under serum-free conditions (column 3). (C) The fold change response of healthy and HD cells to serum-deprivation plus sodium cyanide. None of the fold differences under cyanide conditions were significant.

Multiple differences in gene expression levels occurred between the healthy subjects and HD patients under basal growth conditions (Table 3A). Under basal growth conditions (normal, serum-containing conditions), average mRNA expression for IDH1 (+80.5%), PDHA1 (+20.5%) and SDHC (+25.5%) was higher in the HD patients than in the healthy subjects, whereas DLST expression was reduced (-21.4%) in HD compared to healthy.

Serum-deprivation caused changes in many, but not in all genes (Table 3B-Columns 1–2). Several of the same genes responded similarly to serum-deprivation in lymphoblasts from healthy subjects and HD patients: PDP1 (healthy, +38.5%; HD, +24.7%), PDK4 (healthy, +229.9%; HD, +153.4%), PDK2 (healthy, +63.9%; HD, +85.3%), IDH1 (healthy, +111.8%; HD, +65%) and SDHC (healthy, +46%; HD, +42.1%). Some genes responded uniquely between the two groups to serum-deprivation: PDHA2 (healthy, +70.1%), IDH2 (healthy, -26.8%), IDH3A (healthy, -24%), IDH3G (HD, +28.9%) and TKT (HD, +38.1%).

In serum-free conditions, robust differences occurred between the cells from HD patients and healthy subjects: PDHA1 (+27.3%), IDH1 (+40.5%), SDHB (+31.2%) and SDHC (+21.4%) expressions were higher in HD patients on average than in healthy subjects under serum-free conditions (Table 3B-Column 3).

Cyanide treatment equalized the differences between healthy and HD mRNAs that were found in both basal and serum-free conditions (Table 3C-Column 3). The differences between healthy and HD mRNA expressions that were identified under basal and serum-free conditions were minimized to approximately zero for PDHA1, IDH1, DLST, SDHB and SDHC.

Few mRNAs changed in response to cyanide (Table 3C). Some of the responses of the cells from HD patients and healthy subjects to cyanide were similar. PDK4 (healthy, -47.7%; HD, -32.4%) and PDK2 (healthy, -28.4%; HD, -27.8%) responded to the hypoxia stress similarly in both groups. PDHA2 expression uniquely increased by 33.5% in the healthy lines in response to hypoxia. Strikingly, IDH1 mRNA, which was consistently expressed at higher levels in HD cell lines under basal serum conditions, was uniquely susceptible to cyanide-induced stress in HD: IDH1 expression significantly decreased by 47.9% in the HD lines in response to cyanide. Surprisingly, no differences were found between healthy subjects and HD patients under serum-free plus cyanide conditions. (Table 3C-Column 3).

### Select enzymes show unique activity changes after oxidative stress in healthy and HD cell lines

**Fig 2:**
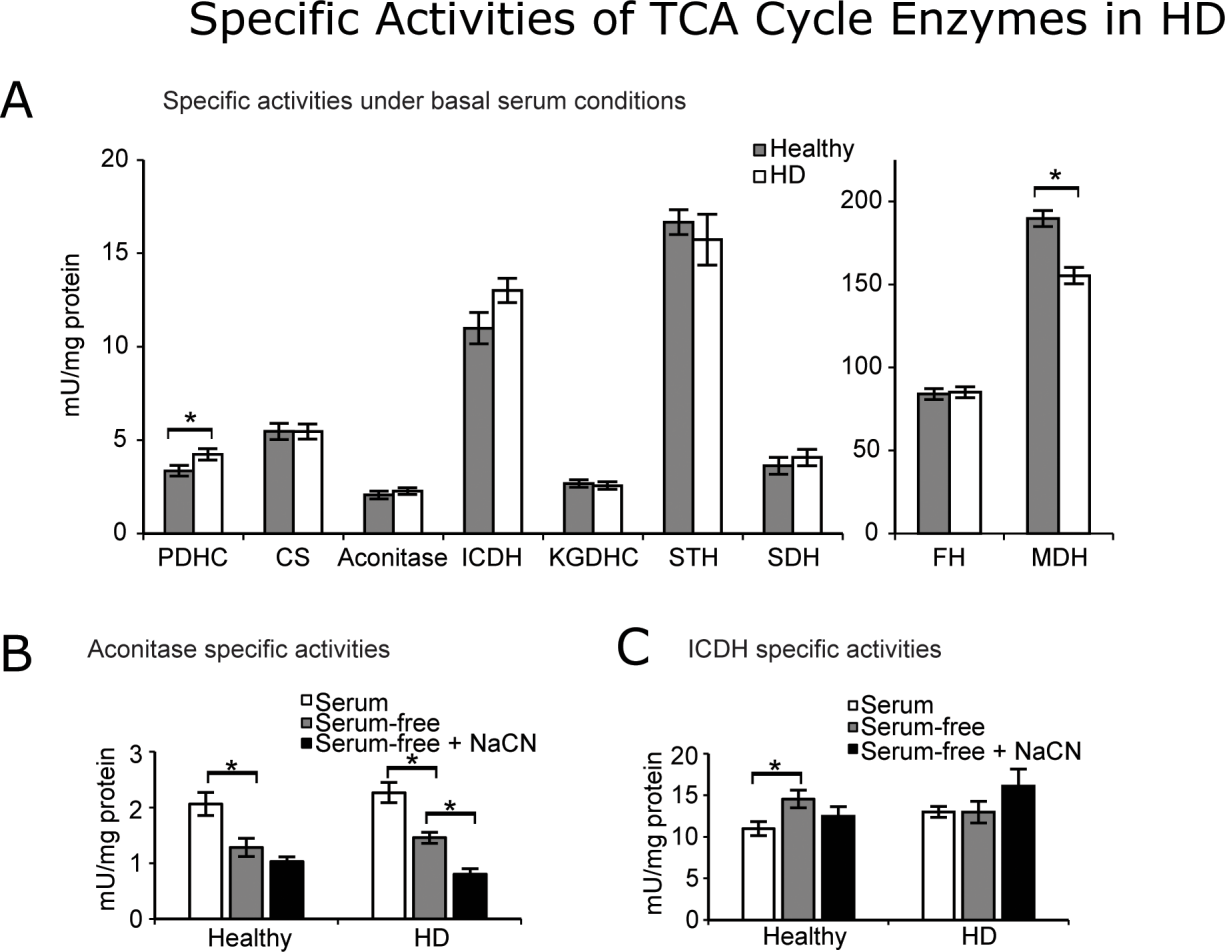
Specific activities of the eight major enzymes of the TCA cycle plus the pyruvate dehydrogenase complex. (A) The evaluations are under basal serum conditions at the 54 hour time point. Each cell line was measured in triplicate in a 96-well plate. Each data point is the mean ± SEM of n = 8 subjects. The differences between the healthy and HD lines under basal serum conditions were compared. (B) Aconitase activity was drastically reduced in the presence of oxidative stressors in both healthy and HD cell lines. (C) Serum-deprivation increased ICDH activity in healthy cells but not in HD cells. * p≤0.05 determined by Student t-test.

Small differences in enzyme activities between healthy controls and HD were apparent when lymphoblasts were incubated with serum (Fig 2A). PDHC activity was 26.3% higher in the HD lines than in the healthy lines in serum. Conversely, MDH activity was 18% lower in HD lines than in healthy lines in serum.

The serum-deprivation stress elicited similar changes in lines from HD patients and healthy subjects in aconitase (Fig 2B) and different responses in ICDH activity (Fig 2C). In response to serum-deprivation, aconitase activity declined by 38% in lines from healthy subjects and by 36% in lines from HD patients. On the other hand, the response of ICDH was quite different (healthy, +32%; HD, -0.3%). The remaining enzymes were relatively resistant to serum-deprivation (S1 Fig).

Aconitase and ICDH were also the most sensitive to serum-deprivation plus cyanide (Fig 2B and 2C). PDHC, CS, STH, FH and MDH did not change by more than 8% in either direction. KGDHC (healthy, -12.3%; HD, -3.4%) and SDH (healthy, +10%; HD, +23.2%) activities showed similar but insignificant changes. Aconitase activity decreased in both healthy (-19.8%) and HD (-44.8%) lines; however, only the decrease in HD was statistically significant. The only enzyme that displayed opposite trends between the subject groups after cyanide was ICDH. ICDH activity decreased in cells from healthy subjects (-14.2%) but increased in cells from HD patients (+24.2%). This difference in response to hypoxia between healthy and HD cells was statistically significant.

### Western blots revealed different protein levels of MDH but not PDHC or ICDH between healthy and HD cell lines

**Fig 3:**
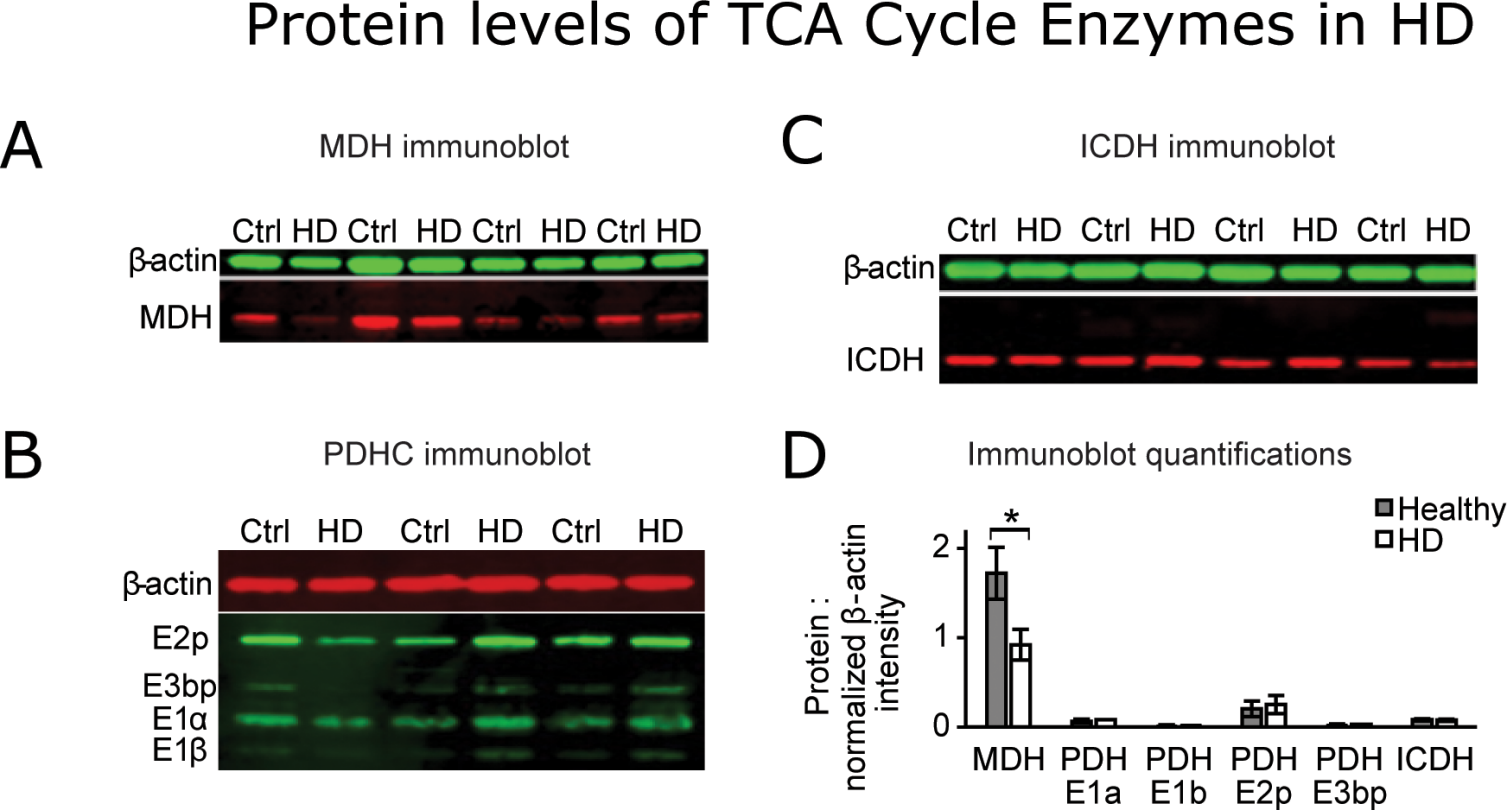
Western blot analysis. (A) A typical MDH Western blot is shown. Four arbitrarily chosen healthy and four HD lines were measured using 7.5 μg of lymphoblast lysate per well. MDH protein was reduced in the HD lines by 47% (p≤0.05). (B) A typical PDHC Western blot is shown. Three arbitrarily chosen healthy and three HD lines were measured using 15 μg of lymphoblast lysate per well. The Western blot was repeated with three different sets of cell lines with similar results (not shown). (C) A typical ICDH Western blot is shown. Seven healthy and seven HD lines were measured using 7.5 μg of lymphoblast lysate per well (4 cell lines for each sample group are shown). (D) Quantification of the measured protein levels is provided. The intensities of the β-actin bands were normalized to the highest intensity. Then the intensities of the specified protein bands were divided by the normalized intensities of the β-actin bands for each respective measurement. Differences were assessed by comparing the ratio of the specified protein to normalized β-actin for each cell line. The MDH and ICDH blots were repeated using the same cell lines (not shown). The final ratios for each cell line and the repeated measurements were averaged and compared using a Student’s t-test. Error bars represent SEM. * p≤0.05 determined by Student t-test.

Protein levels of select enzymes that showed changes or differences in activities between healthy and HD lines were measured by Western blot. Significance was evaluated for each treatment within sample groups and also between healthy and HD cells under each treatment condition. No differences or changes were found in aconitase protein levels (data not shown). MDH protein was 47% lower in HD lines compared to healthy lines under basal serum conditions (Fig 3A). The PDH complex subunits were nearly identical between healthy and HD cells in serum (Fig 3B). ICDH protein levels were similar in HD and in healthy cells under basal serum conditions (Fig 3C). Quantified protein levels normalized to β-actin were calculated as described in the ‘Calculations for Western blots’ section (Fig 3D).

## Discussion

The goal of these studies was to test whether changes in the TCA cycle may provide a link between the gene defect and the pathophysiology of HD. The underlying causes of mitochondrial dysfunction in HD are not fully understood. The alterations to the TCA cycle in HD that were identified by this exploratory study should help focus future studies. Differences in cultured cells from HD patients and healthy subjects are not secondary to a variety of factors (e.g., neurodegeneration or drugs) that confound studies of tissues from patients, and still maintain the advantage of containing the patients’ genetic backgrounds. Changes in MDH and ICDH were particularly striking and unexpected. The results of this study may open avenues for further exploration of TCA cycle enzymes.

Conditions were established to mildly stress the cells to reveal differences between lines from HD patients and healthy subjects. Previous studies showed that lymphoblasts from HD patients could be stressed with cyanide and other oxidants [29]. We exacerbated stress levels by removing serum from the culture media before adding sodium cyanide. Although the serum-deprivation insult produced similar cell death in HD and healthy lines, cyanide reproducibly induced further cell death in healthy but not HD lines. This finding demonstrates that lymphoblast cells from healthy control subjects and those from HD patients respond differently to oxidative stress.

HD cells show markedly reduced reliance on oxidative phosphorylation compared to healthy cells [8]. Our findings of cyanide induced changes in mRNA expression in healthy cells, but not in HD cells, support this idea. Cyanide, which inhibits complex IV of the oxidative phosphorylation chain, shifted mRNA levels of TCA cycle-related genes in healthy cells to the same levels as in cyanide-treated HD cells. Therefore, cyanide-treatment mimics an HD-like cellular response in TCA cycle-related genes in healthy cells. Thus, blocking oxidative phosphorylation had a weaker effect on HD cells than on normal, healthy cells, accounting for the difference in cell death in response to cyanide treatment.

Measurements of mRNAs, protein levels and activities for all of the TCA cycle allow us to draw unique conclusions about the role of the TCA cycle in HD. Message levels for the genes associated with the TCA cycle have not been previously reported for HD peripheral cells or human brains. Indeed, we recently demonstrated they are too unstable to measure in autopsy brains [19]. Changes in mRNA may not be reflected at the protein level because of timing or alterations in protein turnover (e.g., increased proteolysis) or even post-transcriptional changes in the mRNA. The mRNA changes and alterations in enzyme specific activity support the suggestion that PDHC, aconitase and SDH are particularly important in the pathology of HD-related metabolic dysfunction. These measures of mRNA suggest for the first time that ICDH is likely important in HD pathology.

The current studies further support the suggestion that PDHC plays a critical role in the pathophysiology of HD. PDHC provides the acetyl CoA from pyruvate that initiates the TCA cycle. PDHC is composed of multiple subunits and is controlled by inhibitory kinases and activating phosphatases that continuously regulate its activity [30]. PDHC is diminished in autopsy brains of patients that died with HD [15,31]. The activation state of PDHC is also diminished in the R6/2 mouse, an accelerated model of HD, and the PDHC activator dichloroacetic acid is beneficial [32]. However, PDHC activity is higher in brains of the Q175 mouse model of HD [19]. Similarly, the current studies found under basal serum conditions that PDHC activity was increased by 26% in HD compared to control. PDHA1 mRNA expression was also increased in HD compared to healthy lines. Together, these findings indicate that elevation of PDHC may be important in HD pathophysiology, contrary to the long-standing belief that PDHC is compromised in HD. Alternatively, PDHC is compromised by excessive oxidative stress in HD.

Our findings also provide an explanation for the long pursuit of diminished aconitase activity in HD brains. Aconitase activity decreases in the caudate, putamen and cortex of human brains [18], and is decreased in striatum and cortex of R6/2 mice [33], but increased by 32% in cortex of the Q175 mouse model of HD. In this current study, there was no difference in aconitase activity between HD and healthy cells under basal conditions. However, the activities of both declined similarly after serum-deprivation. Cyanide further depressed activities in both sets of lines, and the reduction was significantly greater in the HD lines. The more dramatic decrease after cyanide treatment in HD compared to healthy indicates that aconitase is more vulnerable to hypoxic conditions in HD. Furthermore, aconitase protein was unaffected as measured by Western blot (data not shown). Therefore, the marked reductions with the stressors suggests that the reported decline in aconitase in HD and models of HD is secondary to other factors such as oxidative stress.

The role of ICDH is poorly documented in HD. We did not find a difference in HD autopsy brains [19], although it is diminished in autopsy brains in Down’s syndrome [34] and Alzheimer’s disease [35]. The measures of mRNA expression and enzyme activities suggest that ICDH may be important to HD pathology. IDH1, an isoform found specifically in the cytosol, and IDH2, the mitochondrial isoform, catalyze the conversion of isocitrate to α-ketoglutarate, generating NADPH in the process. NADPH is required for the regeneration of glutathione, which plays a major role in eliminating ROS [36]. Although the pentose shunt is also critical for generating NADPH, differences between HD and healthy cells in transketolase mRNA expression were not observed in serum. Therefore, oxidative stress is likely to alter NADPH production by impacting ICDH activity. Serum-deprivation caused a significant increase in ICDH activity in healthy cells but no change in HD cells. Healthy and HD cells diverged in their responses to hypoxia. Elevated ICDH activity may serve as a protective measure against oxidative stress by generating more NADPH to reduce ROS levels. HD cells notably were unable to activate a protective response by increasing ICDH activity after serum-deprivation. However, this difference is difficult to interpret at the protein level because ICDH is present in both mitochondrial and cytosolic forms that cannot be distinguished by activity measurements or Western blots.

The ICDH isoforms can be distinguished at the mRNA level. The changes in mRNA also suggest that ICDH differs between HD and controls. IDH1 mRNA expression was higher under basal growth and serum-free conditions in the HD compared to healthy cells. Elevated IDH1 mRNA was consistently found in every tested HD patient cell line. Furthermore, cyanide stress uniquely caused nearly a 50% decrease in IDH1 expression in the HD but not healthy cells. It appears that severe mitochondrial stress induced by cyanide eliminates a protective, elevated IDH1 response in HD cells. IDH1 was also elevated 57% in HD Q175 mouse cortex compared to control. Future studies would benefit from genetically manipulating IDH1 and IDH2 levels in striatal neurons and HD mouse models as potential therapies against oxidative stress in HD.

IDH1 mRNA levels may also help serve as a biomarker of metabolism in future clinical trials. The role of malate dehydrogenase in neurodegeneration is not known. MDH is critical in the TCA cycle and also in transferring reducing equivalents across the mitochondrial membranes as part of the malate-aspartate shuttle. MDH increases by 54% in brains from patients that died with Alzheimer’s disease [35]. However, MDH activity was 18% lower in HD lines than in controls under basal serum conditions. MDH Western blots also revealed a greater than 50% decrease in MDH protein in HD under resting conditions. The results are difficult to interpret because MDH, like ICDH, is both cytosolic and mitochondrial. Further clarification of the results will require genetic manipulation of the subunits.

The role of KGDHC in neurodegeneration is well-documented [37]. One preliminary study in the putamen of brains from controls and HD patients suggests that KGDHC activity is reduced [16]. E. coli with 62 CAG repeats exhibit significantly impaired KGDHC activity compared to controls [38,39]. DLST (dihydrolipoyl succinyl transferase) is one of the three subunits of the KGDHC. DLST^+/-^ mice have been shown to be susceptible to mitochondrial toxins. Specifically, treatment with 3-NP and malonate, two toxins that mimic the striatial lesions seen in HD in rats and primates, in these DLST-deficient mice created 2-to-4-fold larger lesions than in controls [40]. We report a 21% decrease in DLST mRNA that extends these impairments to human cells. KGDHC has been reported to decrease after blocking complex II with 3-NP in mice [16], but inhibiting complex IV with cyanide had weak effects in lymphoblasts from healthy and HD patients The lack of change in KGDHC activity under the control conditions or following the two stressors suggests that the cells are able to compensate.

No changes after treatment or differences between the two subject groups were found in several enzymes. CS is reported down in HD putamen and cortex [18], but no significant changes were found in lymphoblasts after either treatment. STH activity, which is poorly documented in HD studies, did not change in healthy and HD cells after either treatment. This finding is similar to a study that also found no change in STH activity in AD [35]. Little to no change was found in SDH activity after either stress. SDH activity is significantly impaired in caudate nucleus from HD patients [41]. Chronic treatment with 3-nitropropionic acid (3-NP), an irreversible inhibitor of SDH (complex II of the oxidative phosphorylation chain), has been shown to cause HD-like striatal degeneration and motor deficits in rodents and primates [23,42]. The lymphoblast findings are not surprising because cyanide selectively inhibits complex IV, thus leaving complex II unaffected. Fumarase activity was shown to be unaffected in the caudate and putamen of human brains [15]. The lack of change in the human lymphoblasts reinforces the existing fumarase literature.

## Conclusion

The experiments utilize the advantages of lymphoblasts to reveal new insights about HD. The large quantity of homogeneous cell populations permits multiple dynamic measures to be made on exactly comparable tissues. The results suggest that the mHTT gene mutation produces particularly striking changes in PDHC, aconitase, ICDH and MDH. IDH1 mRNA may serve as a biomarker of metabolism in future clinical trials. Genetic manipulations of regulatory PDHC kinases and phosphatases, as well as manipulation of cytosolic and mitochondrial isoforms of ICDH and MDH, may prove to be therapeutically beneficial targets in HD.

## Supporting Information

S1 Fig Specific activities of the eight major enzymes of the TCA cycle plus the pyruvate dehydrogenase complex under all treatment conditions. The evaluations are under serum, serum-free, and serum-free + cyanide conditions at the 54 hour time point. Each cell line was measured in triplicate in a 96-well plate. Each data point is the mean ± SEM of n = 8 subjects. The change in response to serum-deprivation and to serum-deprivation plus cyanide for each cell line was assessed for significance. The differences between the healthy and HD lines under basal serum, serum-free, and serum-free plus cyanide conditions were also compared. In the cases of dramatic differences in the responses by the healthy and HD cells, as seen in aconitase cyanide and ICDH serum-deprivation treatments, this difference in response was also evaluated using a Student’s t-test at p≤0.05. The response to cyanide treatment was significantly different between healthy (-19.8%) and HD (-44.8%) cells for aconitase. Additionally, the response to serum-deprivation was significantly different between healthy (+32.3%) and HD (-0.3%) cells for ICDH.* p≤0.055 for difference between HD and healthy under basal serum differences for PDHC. * p≤0.05 for difference between HD and healthy under basal serum differences for MDH. * p≤0.05 for healthy serum-deprivation response for aconitase. Ω p≤0.05 for HD serum-deprivation response for aconitase. Ѱ p≤0.05 for HD response to hypoxia for aconitase. ¥ p≤0.05 for healthy serum-deprivation response for ICDH.
(TIF)

## Abbreviations

CS: citrate synthase
ETC: electron transport chain
FH: fumarate hydratase
HD: Huntington’s disease
ICDH: isocitrate dehydrogenase
KGDHC: α-ketoglutarate dehydrogenase complex
MDH: malate dehydrogenase
PBS: Phosphate buffered saline
PDHC: pyruvate dehydrogenase complex
SDH: succinate dehydrogenase
STH: succinyl thiokinase
TCA cycle: tricarboxylic acid cycle
TKT: transketolase
