# Supplementary figures and images for: Novel Metabolic Abnormalities in the Tricarboxylic Acid Cycle in Peripheral Cells From Huntington’s Disease Patients
Source: PLoS One. 2016 Sep 9;11(9):e0160384. doi: 10.1371/journal.pone.0160384 (PMC5017661; doi:10.1371/journal.pone.0160384)

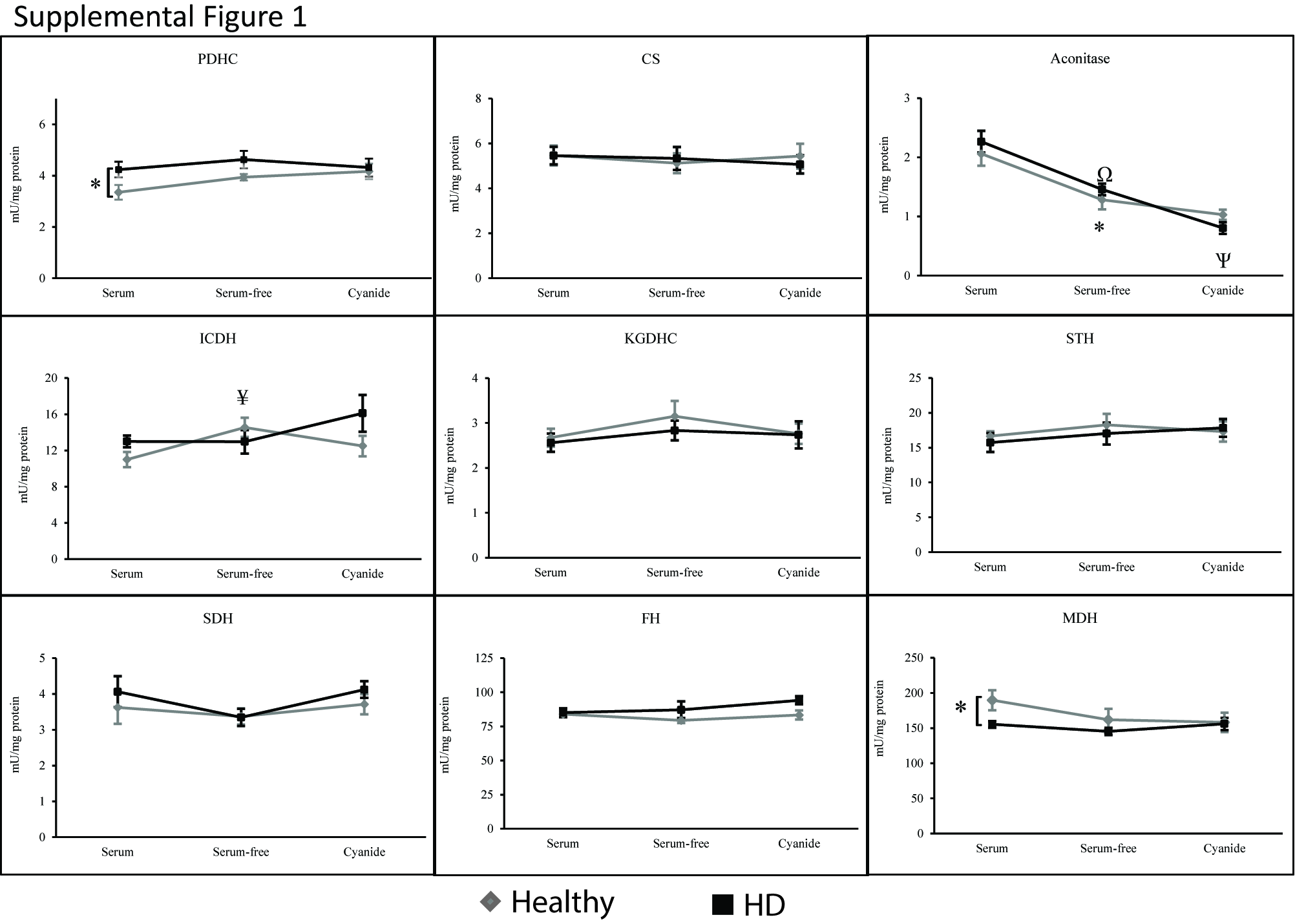

Supplement: S1 Fig — The evaluations are under serum, serum-free, and serum-free + cyanide conditions at the 54 hour time point. Each cell line was measured in triplicate in a 96-well plate. Each data point is the mean ± SEM of n = 8 subjects. The change in response to serum-deprivation and to serum-deprivation plus cyanide for each cell line was assessed for significance. The differences between the healthy and HD lines under basal serum, serum-free, and serum-free plus cyanide conditions were also compared. In the cases of dramatic differences in the responses by the healthy and HD cells, as seen in aconitase cyanide and ICDH serum-deprivation treatments, this difference in response was also evaluated using a Student’s t-test at p≤0.05. The response to cyanide treatment was significantly different between healthy (-19.8%) and HD (-44.8%) cells for aconitase. Additionally, the response to serum-deprivation was significantly different between healthy (+32.3%) and HD (-0.3%) cells for ICDH.* p≤0.055 for difference between HD and healthy under basal serum differences for PDHC. * p≤0.05 for difference between HD and healthy under basal serum differences for MDH. * p≤0.05 for healthy serum-deprivation response for aconitase. Ω p≤0.05 for HD serum-deprivation response for aconitase. Ѱ p≤0.05 for HD response to hypoxia for aconitase. ¥ p≤0.05 for healthy serum-deprivation response for ICDH. (TIF) [file pone.0160384.s001.tif]
